# Therapeutic Interaction of Systemically-Administered Mesenchymal Stem Cells with Peri-Implant Mucosa

**DOI:** 10.1371/journal.pone.0090681

**Published:** 2014-03-20

**Authors:** Ryosuke Kondo, Ikiru Atsuta, Yasunori Ayukawa, Takayoshi Yamaza, Yuri Matsuura, Akihiro Furuhashi, Yoshihiro Tsukiyama, Kiyoshi Koyano

**Affiliations:** 1 Section of Implant and Rehabilitative Dentistry, Division of Oral Rehabilitation, Faculty of Dental Science Kyushu University, Fukuoka, Japan; 2 Department of Molecular Cell Biology and Oral Anatomy, Graduate School of Dental Science Kyushu University, Fukuoka, Japan; French Blood Institute, France

## Abstract

**Objectives:**

The objective of this study was to investigate the effect of systemically transplanted mesenchymal stem cells (MSCs) on the peri-implant epithelial sealing around dental implants.

**Materials and Methods:**

MSCs were isolated from bone marrow of donor rats and expanded in culture. After recipient rats received experimental titanium dental implants in the bone sockets after extraction of maxillary right first molars, donor rat MSCs were intravenously transplanted into the recipient rats.

**Results:**

The injected MSCs were found in the oral mucosa surrounding the dental implants at 24 hours post-transplantation. MSC transplantation accelerated the formation of the peri-implant epithelium (PIE)-mediated mucosa sealing around the implants at an early stage after implantation. Subsequently, enhanced deposition of laminin-332 was found along the PIE-implant interface at 4 weeks after the replacement. We also observed enhanced attachment and proliferation of oral mucous epithelial cells.

**Conclusion:**

Systemically transplanted MSCs might play a critical role in reinforcing the epithelial sealing around dental implants.

## Introduction

Dental implant therapy is one of the most important and effective prosthodontic therapy options for partially and completely edentulous patients. Dental implants based on the concept of “osseointegration”, a term explaining the fixation of a titanium implant in the bone [1], have resulted in dramatic therapeutic success and clinical improvement. However, the peri-implant tissue is always exposed to the possibility of inflammation because the titanium body penetrates the surrounding oral mucosa. Although the mucosal structure around the dental implant shows similarities to normal/healthy gingiva with innate defense mechanisms [2]–[4], many researchers have described the biological weakness of the peri-implant epithelium (PIE)-mediated sealing against the oral environment [5], [6]. Therefore, improvement of the tight PIE-mediated sealing around dental implants is strongly desired to enable clinical success and improve outcomes for oral implant therapy.

Mesenchymal stem cells (MSCs) were first identified as postnatal stem cells in bone marrow by Friedenstein and colleagues [7], and were subsequently found in several human tissues, including adipose tissue, the umbilical cord, and dental pulp [8]–[10]. Recently, the minimum criteria to define MSCs was proposed by the Mesenchymal and Tissue Stem Cell Committee of the International Society for Cellular Therapy [11] as follows: (1) a capacity for adherence; (2) typical immunophenotypes including positivity for CD105, CD73, and CD90, and negativity for CD45, CD34, CD14, and CD11b; (3) multipotency including cell types of at least three lineages, such as osteoblasts, chondroblasts, and adipocytes. Furthermore, MSCs exhibit anti-inflammatory functions toward diverse immune cell types including lymphocytes, macrophages, and natural killer cells [12]. Therefore, many researchers have a great deal of interests in the therapeutic potential of human MSCs to treat a variety of human diseases [13], [14].

In this study, it was investigated that the MSCs potential was applied for implant treatment with some troubles, delayed healing and mucosa inflammation based on the low sealing around implant. A few studies have reported that epithelial healing after implant placement is very similar to mucosa wound healing [15]. Wound healing progresses through a genetically programmed repair process that involves inflammation, cell proliferation, re-epithelialization, formation of granulation tissue, angiogenesis, interactions between various cells, and matrix and tissue remodeling [16]. Additionally, bacteria can accumulate around the implant circumference and induce inflammatory destruction more easily than around the natural tooth [17]. Under such abnormal situations, the PIE structure is formed along the implant surface. In all situations, the aim of treatment is to provide soft tissue regeneration to restore the structure, function, and physiology of the damaged tissues. Thus, it is critical to stabilize the epithelial soft tissue seal by promotion of epithelial cell adherence [18].

The relationship between MSC-based therapy and PIE-implant interface sealing is not well understood. The hypothesis of the present study was that systemic MSCs accumulate around the implant in the early stage and promote PIE formation and soft tissue attachment to the implant surface.

## Materials and Methods

### 1. Animals

Male Wistar rats (4- and 6-weeks-old) and GFP-transgenic SD-Tg (CAG-EGFP) rats were purchased from Kyudo Lab (Tosu, Japan) and Japan SLC (Shizuoka, Japan), respectively. These animal experiments were performed under an institutionally approved guideline for animal care established by Kyushu University (approval number: A24-237-0).

### 2. Isolation and culture of MSCs

MSCs were isolated from the bone marrow of Wistar or GFP-transgenic rats based on a colony forming unit-fibroblast (CFU-F) assay [19]. Briefly, bone marrow cells were flushed out of the bone cavities of rat femurs and tibias, and then treated with a 0.85% NH_4_Cl solution for 10 minutes to lyse red blood cells. The cells were passed through a 70-µm cell strainer to obtain a single cell suspension. The single cells were seeded at 1×10^6^ cells/dish in 100-mm culture dishes. At 1 day after seeding, the cells were washed with phosphate buffered saline (PBS) and cultured in growth medium consisting of alpha minimum essential medium (Invitrogen, Grand Island, NY) containing 20% fetal bovine serum (Equitech-Bio, Kerrville, TX), 2 mM L-glutamine (Invitrogen,), 55 µM 2-mercaptoethanol (Invitrogen), 100 U/ml penicillin, and 100 µg/ml streptomycin (Invitrogen). After 1 week of culture, the CFU-Fs had formed colonies. The adherent mesenchymal cells in these colonies (referred to as “MCs” hereafter) were detached by trypsin, reseeded as new cultures, and expanded for further studies.

### 3. CFU-F assay

The CFU-F assay was performed as described previously [20]. Passage one MSCs were seeded at appropriate cell numbers in 100-mm dishes (Nalge Nunc, Rochester, NY). After 16 days, the cells were stained with a mixture of 0.1% toluidine blue (Merck, Darmstadt, Germany) and 2% paraformaldehyde (PFA; Merck) solution. Clusters containing >50 cells were considered as colonies. Total colony numbers were counted per dish. The CFU-F assay was repeated in independent experiments.

### 4. Immunofluorescence

Passage two MSCs (2×10^4^ cells/dish) were seeded on 35-mm dishes and incubated for 12 hours at 37°C under 5% CO_2_. Then, the slides were fixed in 4% PFA for 5 minutes and blocked with normal serum matched to the secondary antibodies for 1 hour followed by incubation with the mouse anti-rat CD44, CD90, and CD105 antibodies (1∶100, Sigma–Aldrich) overnight at 4°C. Then, the slides were treated with FITC-conjugated secondary antibodies (1∶200, Jackson Immuno Research, West Grove, PA) for 1 hour at room temperature (RT) and mounted with VECTASHIELD Mounting Medium containing 4′6-diamidino-2-phenylindole (DAPI) (Vector Laboratories, Burlingame, CA).

### 5. Flow cytometric analysis of cell surface markers

Passage two MSCs were collected and incubated with mouse anti-rat CD44, CD90, and CD105 antibodies (2 µg/ml, Chemicon International, Temecula, CA) for 60 minutes at 4°C and then an allophycocyanin-labeled secondary antibody (2 µg/ml, Vender, City, State/Country) for 30 minutes at 4°C. The analysis was then carried out using a FACS Calibur system (BD Biosciences) [21].

### 6. Osteogenic differentiation assay

Passage two MSCs (5×10^5^ cells/dish) were grown on 35-mm dishes to confluency in growth medium and then cultured in osteogenic culture medium [growth medium containing 1.8 mM KH_2_PO_4_ (Sigma-Aldrich, St. Louis, MO) and 10 nM dexamethasone (Sigma-Aldrich)]. After 28 days of osteogenic induction, the cultures were stained with a 1% Alizarin Red S solution (Sigma-Aldrich). The expression of osteogenic markers including alkaline phosphatase (ALP), Runx2 and osteocalcin (OCN) was determined by western blot analysis.

### 7. Adipogenic differentiation assay

Passage two MSCs (5×10^5^ cells/dish) were grown on 35-mm dishes to confluency in growth medium and then cultured in adipogenic culture medium [growth medium containing 0.5 mM isobutylmethylxanthine (Sigma–Aldrich), 60 µM indomethacin (Sigma–Aldrich), 0.5 µM hydrocortisone (Sigma–Aldrich) and 10 µg/ml insulin (Sigma–Aldrich)]. After 14 days of adipogenic induction, the cultures were stained with Oil Red O. The expression of adipogenic markers including lipoprotein lipase (LPL) and peroxisome proliferator-activated receptor γ (PPARγ) was determined by western blot analysis.

### 8. Chondrogenic differentiation assay with the pellet culture technique

Passage two MSCs (5×10^5^ cells/dish) were collected in 5-ml conical polypropylene tubes and then pelleted for 6 minutes at 1,600 rpm. The cell pellet was incubated in chondrogenic culture medium [growth medium containing 50 µg/ml ascorbic phosphate (Sigma-Aldrich), 2 mM pyruvate (Sigma-Aldrich), 10 mg/ml transforming growth factor-β1 (Sigma-Aldrich), and 10 µg/ml insulin (Sigma-Aldrich)]. After 21 days, the pellets were washed with PBS and then fixed with 4% PFA for 4 hours.

### 9. Western blot analysis

Proteins were separated by sodium dodecyl sulfate polyacrylamide gel electrophoresis on 7.5% gels and transferred to polyvinylidene fluoride membranes (Bio-Rad Laboratories, Hercules, CA). The blots were probed for 24 hours at 4°C with primary polyclonal antibodies against rat osteogenic markers (ALP, Runx2, and OCN) and adipogenic markers (PPARγ and LPL) (all diluted at 1∶100; Cell Signaling, Beverly, MA). The membranes were then incubated with secondary antibody for 60 minutes at RT, and visualized using an ECL Western Blotting Analysis System (GE Healthcare, Little Chalfont, UK).

### 10. Experimental dental implants

**Figure 1:**
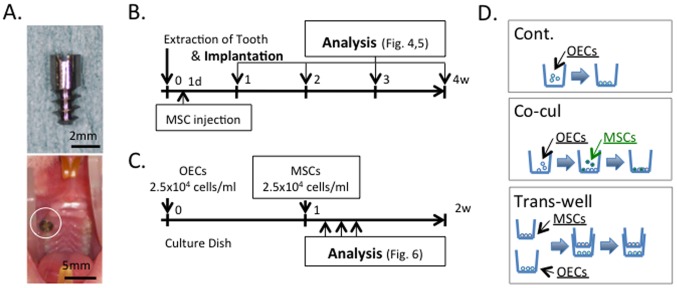
Design of the *in vivo* and *in vitro* experiments. (A) Photograph of the experimental implants (upper panel). Photograph of the implant in the rat oral cavity (lower panel). There is no apparent inflammation in the oral mucosa around the implant. (B) Experimental protocol for the *in vivo* study. Implantation was immediately performed after tooth extraction. Then, 24 hours after implantation, mesenchymal stem cells (MSCs) were injected via the tail vein. The structure of the epithelial tissue around the tooth or implant was observed after 1, 2, 3 and 4 weeks. (C) Experimental protocol for the *in vitro* study. Rat oral epithelial cells (OECs) were analyzed for changes in cell morphology 7 days after seeding OECs with MSCs under various culture conditions. (D) Experimental methods used for the *in vitro* study.

Similar to previously described designs [15], [22], one-piece, screw-type pure titanium implants (Japan Industrial Standards Class 1 equivalent to ASTM grade 1) (Sky blue, Fukuoka, Japan) with machine-polished surfaces were used in this study (Fig. 1A). The roughness of the implant surface was measured with a laser scanning microscope (VK-9710, Keyence, Osaka, Japan) and the arithmetic mean roughness (Ra) was 0.16 µm. Before use, the implants were treated with 100% acetone and distilled water and subsequently sterilized by autoclaving.

### 11. Oral implantation

The oral implantation procedure was completed according to our previous immediate-implantation study [23] (Fig. 1B). Briefly, maxillary right first molar was extracted from 27 male Wistar rats (6-weeks-old, 150–180 g), and the experimental implant was screwed into the cavity under systemic chloral hydrate and local lidocaine hydrochloride (Abbott Laboratory, North Chicago, IL) anesthesia. Following surgery, the rats were administered buprenorphine (0.05 mg/kg, i.m.) to relieve pain.

### 12. MSC transplantation

Passage three MSCs (1×10^6^ cells) were injected into the rats with or without experimental dental implants via the tail vein (n = 5 in each group) at 24 hours post-implantation under anesthesia. As controls, rats received PBS (n = 5) or rat skin-derived fibroblasts (n = 5) in place of MSCs. All rats were sacrificed at 1, 2, 3 and 4 weeks post-transplantation.

### 13. Immunohistochemistry

According to our previous report [22], at the end of each experimental period, rats were deeply anesthetized and perfused intracardially with heparinized phosphate buffered saline, followed by 4% PFA (pH 7.4). The maxillae were dissected and demineralized in 5% tetrasodium ethylenediaminetetraacetate for 4 days at 4°C. The oral mucosa surrounding the implant and tooth site was carefully removed from the bone, implant or tooth, and then immersed in a 20% sucrose solution. The samples were embedded into O.C.T compound (Sakura, Tokyo, Japan) and cut into 10 µm-thick bucco-palatal sections with a cryostat at −20°C. These sections were then immunostained using an avidin-biotin complex (ABC) procedure (Vectastain ABC, Vector Laboratories, Burlingame, CA), as described previously [24], [25]. Briefly, after treatment with 10% normal goat serum for 30 minutes at RT, samples were incubated overnight at 4°C with a rabbit polyclonal anti-rat laminin-332 antibody (Santa Cruz Biotechnology, Santa Cruz, CA), then treated with biotinylated goat anti-rabbit IgG (1∶200) for 45 minutes at RT, and finally reacted with the ABC reagent (1∶100) for 60 min at RT. Immuno-positive reactions were visualized by treatment for 5 minutes in 0.02% 3,3′-diaminobenzidine tetrahydrochloride (Dojin Laboratories, Kumamoto, Japan) and 0.006% H_2_O_2_, at RT and the sections were counterstained lightly with hematoxylin.

### 14. Isolation and culture of oral mucous epithelial cells (OECs)

OECs were cultured according to a previous report [26] (Fig. 1C). Briefly, oral mucosa derived from 4-day-old Wistar rats was incubated with dispase (1×10^3^ IU/ml) in Mg^2+^- and Ca^2+^-free PBS for 12 hours at 4°C. The oral epithelium (OE) was then peeled from the connective tissue using two pairs of tweezers. The epithelium was dispersed by pipetting 10 times and seeded onto dishes. OECs were cultured in defined keratinocyte serum free medium (Invitrogen) containing gentamicin (50 µg/ml) on plastic in a humidified atmosphere of 95% air and 5% CO_2_ at 37°C.

Additionally, to determine the *in vitro* cellular effects of MSCs on OECs, the epithelial cells were co-cultured with MSCs directly or indirectly (Fig. 1D). For direct co-culture, OECs (2.5×10^4^/ml: show the number per well) were first plated followed by MSCs (2.5×10^4^/mL: show the number per well). For indirect co-culture using Transwell plates (BD Biosciences), OECs (2.5×10^4^ per well) were plated in the lower chambers and MSCs (2.5×10^4^ per well) were seeded in the upper chambers.

### 15. Cell adhesion assay

OEC adhesion assays were conducted according to previously published methods [27]. Twenty-four hours after co-culture with OECs and MSCs, non-adherent or weakly attached cells were removed by shaking (3×5 min at 75 rpm) on a rotary shaker (NX-20, Nissin, Tokyo, Japan). Adherent cells were then counted and calculated as a percentage of the initial count, which was used to define the adhesive strength of the cells.

### 16. Cell proliferation assay

Cell proliferation was assayed using cell proliferation kits (Molecular Probes Inc., Eugene, OR). Cultured cells were exposed to 5-bromo-2′-deoxyuridine (BrdU) in culture medium for 1 hour and then fixed in 70% methanol for 30 minutes. Fixed cells were incubated with mouse anti-BrdU monoclonal antibody (1∶100 dilution) for 1 hour and then with FITC-conjugated anti-mouse IgG (Chemicon International; 1∶100 dilution) for 30 minutes.

### 17. Statistical analysis

Data are expressed as the mean ± SD. One-way analysis of variance with Fisher's least significant difference tests was performed. *P*-values of less than 0.05 were considered significant. Experiments were performed using triplicate samples and were repeated three or more times to verify their reproducibility.

## Results

### 1. Isolation and characterization of MSCs from rat bone marrow

**Figure 2:**
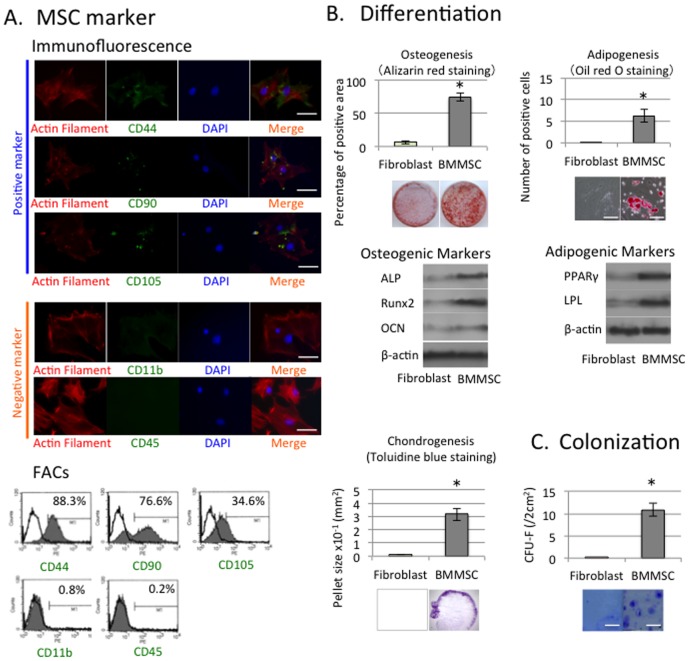
Multipotential differentiation of rat MSCs. (A) Expression of stem cell markers in rat BMMSCs. Cells cultured in 100 mm culture dishes were fixed and immunostained with specific Abs for rat CD-44, CD-90, CD-105 or CD-11b. Cells were incubated with rhodamine- or FITC-conjugated secondary Abs. (A1) Under a fluorescence microscope, positive signals were quantified in five random fields and expressed as the percentage of total DAPI-positive cells (bar = 100 µm) (mean ± SD). (A2) Expression of cell surface markers on MSCs as determined by flow cytometry. (B) (B1) Osteogenic differentiation of MSCs. After culture under osteogenic differentiation conditions for 4 weeks, osteogenic differentiation was determined by Alizarin Red S staining and western blot analysis of specific proteins (ALP, Ranx-2, OCN). The graph shows the quantification of the Alizarin Red S dye content in differentiated osteocytes from independent experiments (mean ± SD). Scale bar, 50 µm. (B2) Adipogenic differentiation of MSCs. After culture under adipogenic differentiation conditions for 2 weeks, adipocyte differentiation was determined by Oil Red O staining and western blot analysis of specific proteins (PPAR-γ, LPL). The graph shows the quantification of the Oil Red O dye content in differentiated adipocytes from independent experiments (mean ± SD). *P<0.05 (C) Single colony-derived rat stem cells represented a putative MSC population with clonogenic renewal properties.

The colony formation rate was 4–6% per bone marrow-derived cell. Immunofluorescence and flow cytometric analyses showed that passage two MCs were positive for CD44, CD90, and CD105, and negative for CD45 and CD11b (Fig. 2A).

Next, when the MSCs were cultured under an osteogenic condition for 4 weeks, the cultures showed accumulation of calcium by Alizarin Red staining. Furthermore, western blotting revealed expression of osteoblast-specific molecules including ALP, Runx2, and OCN (Fig. 2B). Under adipogenic induction conditions for 2 weeks, the MCs were capable of storing intracellular lipid droplets as shown by Oil Red O staining. Additionally, western blotting confirmed expression of adipocyte-specific markers including LPL and PPARγ (Fig. 2B). Finally, the MCs were able to differentiate into chondrocytes using the pellet culture technique (Fig. 2B). These findings indicated that our isolated cell population contained MSCs according to the criteria by the Mesenchymal and Tissue Stem Cell Committee of the International Society for Cellular Therapy [11]. Figure 2C shows that single colony-derived rat stem cells represented a putative MSC population with clonogenic renewal properties.

### 2. Homing of transplanted GFP-labeled MSCs to the surrounding tissues of dental implants (Fig. 3)

**Figure 3:**
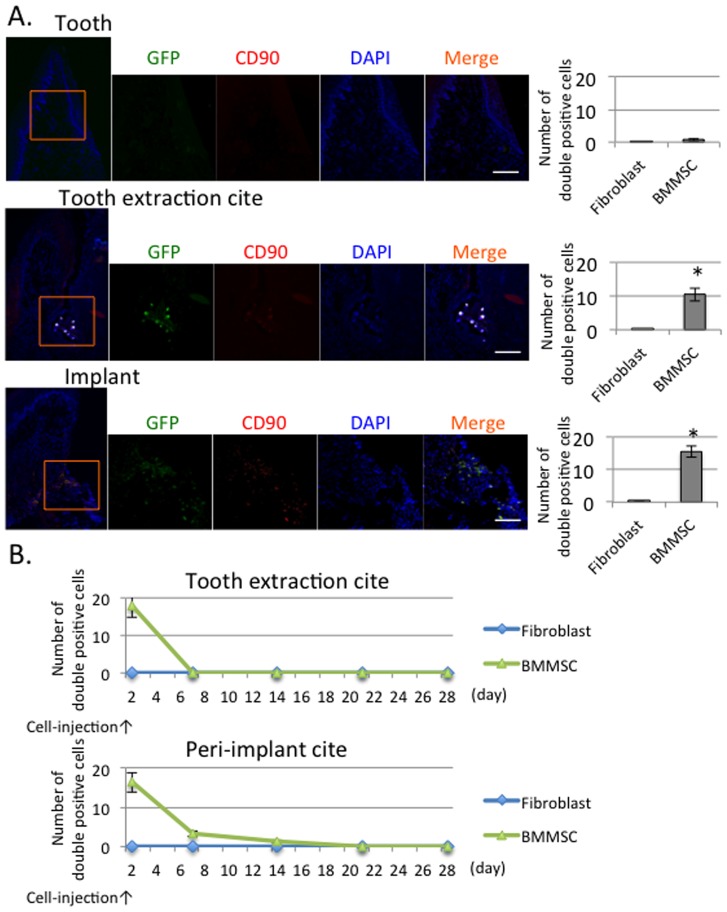
Accumulation of GFP-transgenic injected MSCs after tooth extraction or implantation. (A) Around the experimental implants, injected MSCs selectively accumulated at the extraction site or peri-implant tissue. However, no double-positive MSCs were observed in gingival mucosa. Fibroblasts isolated from back skin as negative controls did not accumulate at any sites. Bar = 100 µm. (B) The accumulated MSCs remained around the extraction and implantation sites for approximately 1 to 2 weeks.

To determine whether transplanted MSCs are able to home to the oral mucosa around dental implants, we intravenously infused bone marrow MSCs derived form GFP-transgenic rats and analyzed their localization in the surrounding mucosa of dental implants at 3 days after infusion. GFP-positive cells were observed in the connective tissues of the peri-implant mucosa beneath the dental implants (Fig. 3A, S1). Interestingly, the localization of GFP-positive MSCs was limited to the connective tissue around the apical portion of the PIE. GFP-positive MSCs were not found in the naïve gingivae around natural teeth. However, GFP-positive MSCs were observed at the wound site after tooth extraction (Fig. 3A). Additionally, these double-positive cells remained around the extraction and implantation site for about 1 to 2 weeks (Fig. 3B). Transplanted fibroblasts isolated from the back skin of GFP-transgenic rats were not found at any site. Almost all injected cells were detected in the lung or peripheral blood (Fig. S2).

### 3. Healing of the oral mucosa around dental implants (Fig. 4, 5)

**Figure 4:**
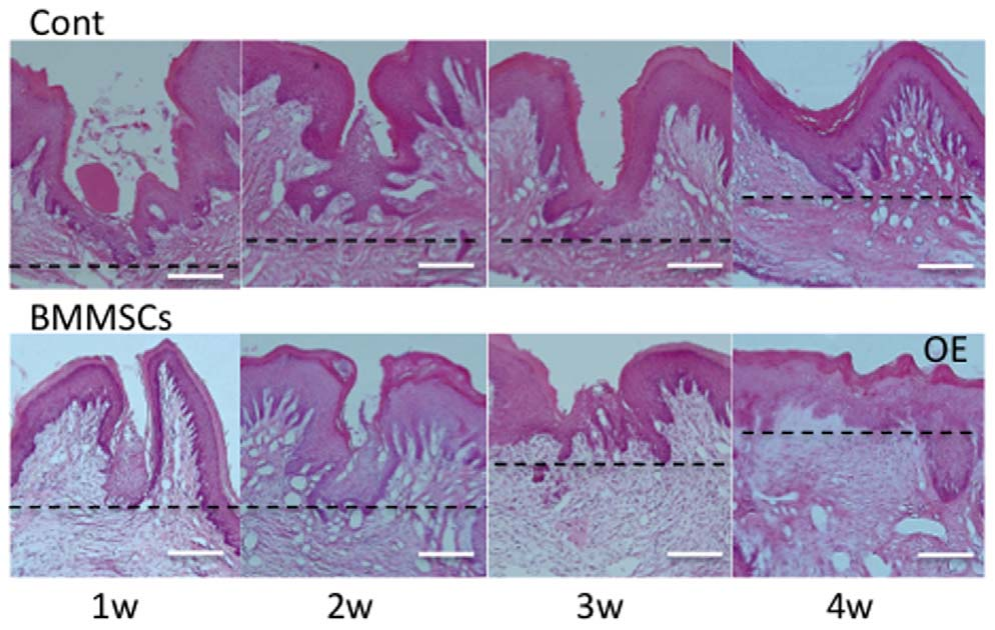
Formation of oral mucosae by injected rat MSCs after tooth extraction. (1w) One week after extraction, a thin epithelial layer extended horizontally on the wound site in the MSC group (B), but not in the control group (A). (2, 3w) After 2 and 3 weeks, in both groups, the OE became mature in structure. In the MSC group, the wound site exhibited the same horizontal height as normal OE. (4w) After 4 weeks, both groups exhibited consolidated OE. Hematoxylin and eosin staining. Bar = 100 µm.

First, to evaluate the effect of MSC transplantation on mucosal repair, we observed the healing process of the oral mucosa at 1, 2, 3, and 4 weeks after tooth extraction (Fig. 4). Interestingly, in the MSC-transplanted group, the healing epithelium bulged vertically and completely covered the extraction cavity at 1 week after the extraction. The regenerated oral epithelium (OE) became more mature until week 4. In contrast, in the non-transplanted group, a thin epithelial layer extended horizontally from the wounded edges of the oral sulcular epithelium (OSE) to the scar at 1 week after the extraction. The regenerating epithelium finally covered the extraction wound. These histological findings indicate that MSC transplantation accelerates the repair process of the oral mucosa after tooth extraction, and that MSC transplantation is capable of inducing the formation of peri-implant mucosa.

**Figure 5:**
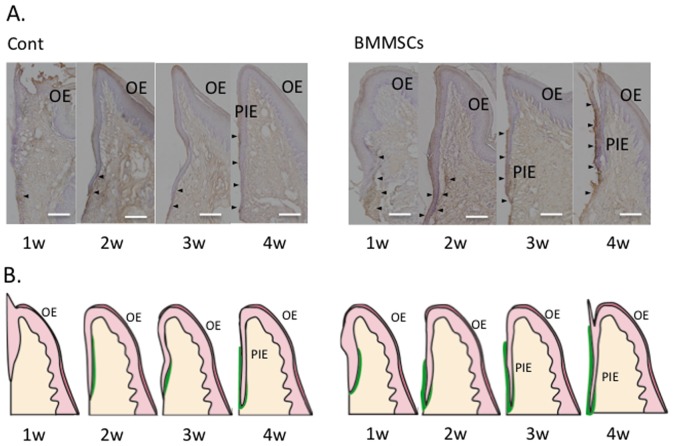
Laminin-332 distribution during the formation of peri-implant epithelium (PIE) following MSC injection. (A) (1w) After 1 week, laminin-332 was expressed in the BM of the new epithelium and in the FC, but not in the epithelial layer facing the implant. After 2 weeks, laminin-332 was intensely expressed in the CT, and the innermost cells of the PIE facing the implant were positive for laminin-332 in the MSC group. (3w) After 3 weeks, a weak positive reaction for laminin-332 was observed in the PIE as a thin band along the implant-PIE interface in the MSC group only. (4w) After 4 weeks, the PIE was completely formed in both groups. Laminin-332 was scarcely expressed along the upper portion of the implant-PIE interface. Laminin-332 was weakly expressed along the BM. Hematoxylin staining. Bar = 100 µm. (B) Lower panels show schematics of these tissue arrangements in the gingiva around the implant. Green lines show laminin-332-positive areas.

Next, we examined the effect of MSC transplantation on formation of the peri-implant mucosa. Rat MSCs were intravenously injected into rats that received dental implants immediately after tooth extraction. Development of the peri-implant mucosa was then analyzed by histochemical and immunohistochemical analyses at 1, 2, 3, and 4 weeks post-implantation (Figs. 4 and 5). In the non-transplanted group, hypertrophic OE/OSE with a corneous layer began to extend along the surface of the dental implant and present at the upper-middle portion of the implant until week 2. Moreover, a thin non-keratinized epithelium had extended from the keratinized epithelium and finally formed non-keratinized PIE and keratinized peri-implant sulcular epithelium (PISE) at week 4 as reported previously [15], [22]. However, the MSC-transplanted group exhibited more accelerated formation of the PIE around the dental implant compared with that in the non-transplanted group (Fig. 5). At week 2, a thin epithelium without keratinization extending from the OSE had spread further along the implant. The non-keratinized epithelium, PIE, and PISE covered the surface of the dental implants at week 3.

Laminin-332 is a component of the basement membrane (BM), which is considered to be involved in migration and adhesion of PIE cells, indicating that laminin-332 plays an important role in PIE formation [19], [22], [24]. To confirm the efficacy of MSC transplantation to promote formation of the PIE around dental implants, we examined the distribution of laminin-332 during the PIE development process (Fig. 5). In the non-transplanted group, laminin-332 was expressed initially along the BM under the OSE and OE in both groups and sparsely in the connective tissue until week 2. At week 3, laminin-332 was strongly expressed in the connective tissues around the apical portion of the immature PIE, but not in the inner interface between the PIE and dental implant. Laminin-332 was also expressed weakly at the BM under the PISE. At week 4, laminin332 was distributed as a band along the implant-PIE interface and PIE-connective tissue interface. However, the MSC-transplanted group showed earlier deposition of lamin-332 around the PIE. Additionally, at week 4, the laminin-332-positive structure at the PIE-implant interface extended to a more upper portion compared with that in the non-transplanted group. Furthermore, the laminin-332-positive structure at the PIE-implant interface in the MSC-transplanted group showed stronger laminin-332 expression than that in the non-transplanted group.

### 4. Direct and indirect interaction between co-cultured MSCs and OECs (Fig. 6)

**Figure 6:**
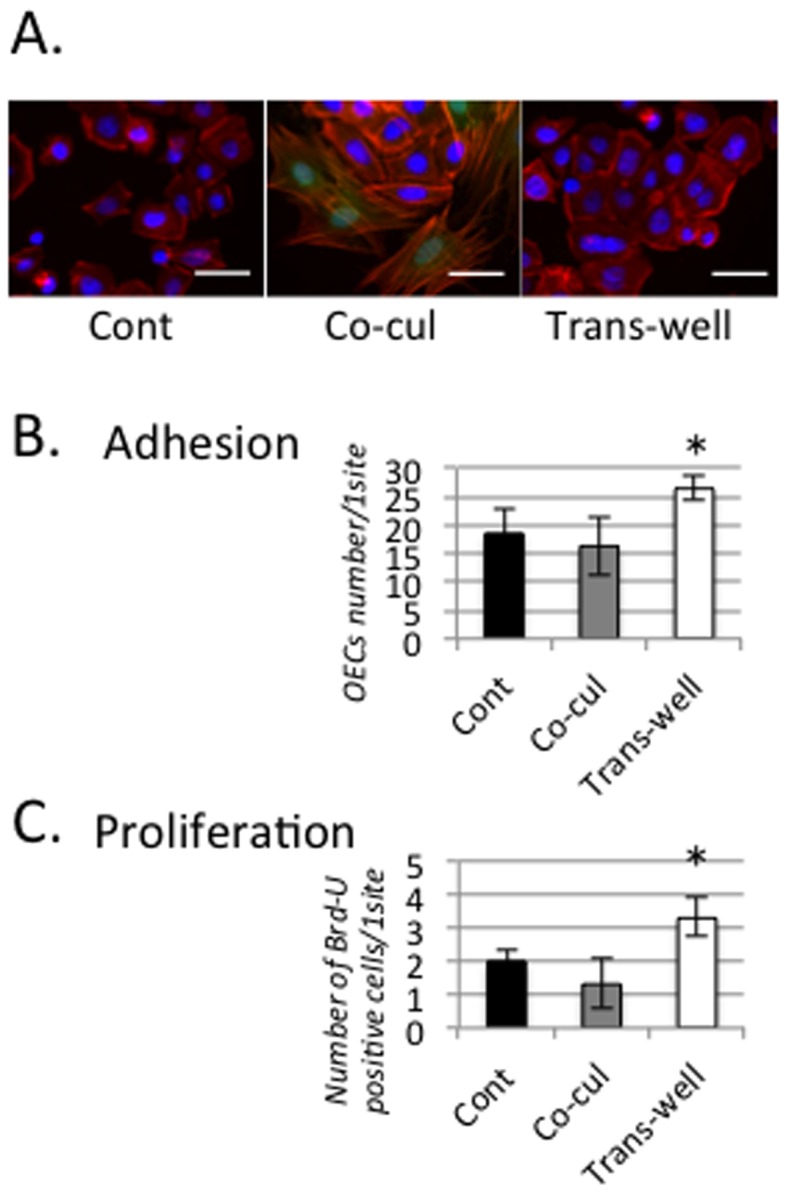
Relationship between MSCs and OECs in co-culture. (A) Rate of proliferation as determined by Brd-U assay. The rate of OEC proliferation in the trans-well condition was significantly lower than in cells grown with C-medium, but the proliferation rate was markedly increased by indirect culture with MSCs. (bar = 100 µm) (B) Apoptosis analysis by FACS. Apoptosis of OECs was detected and quantified by FACS after annexin V and 7AAD staining. The lower table was arranged from the upper data. The rate of apoptosis in OECs in the Co-cul condition was much higher than in the trans-well condition. Each data point represents the mean ± SD of two parallel experiments. #; *P*<0.05 versus trans-well.

OECs were detected and quantified by adhesion and proliferation assays after 24 hours in co-culture with or without MSCs in co-culture and transwell groups (Fig. 6A). Many OECs adhered when cultured with MSCs indirectly in transwells, but fewer cells adhered when co-cultured directly with MSCs (Fig. 6B). OEC proliferation was significantly higher in the indirect co-culture in transwells than that in direct co-culture (Fig. 6C).

## Discussion

### 1. Isolation and characterization of MSCs from bone marrow (BMMSCs)

A variety of adult stem cells and/or precursor cells have been reported in several complex tissues or organs, including the dental tissues; however, few studies have confirmed which population of precursor cells exists in rat MSCs. In this study, MSCs from rat gingival tissue displayed CD44, CD90 and CD105, but were negative for CD11b and CD45, as reported previously [28]. To validate our method of rat BMMSC isolation, Figure 2 shows that the cells isolated from the bone marrow of healthy donor rats had CD44 (88.3%), CD90 (76.6%), and CD105 (34.6%) positive signals by immunofluorescence and FACS (Fig. 2A), thus validating the MSC isolation method.

In this study, we used colony formation assays to show the presence of cells exhibiting the functional capacities of MSCs as described elsewhere [20], [29]. Phenotypically defined rat MSCs adhered to culture dishes and 4–6% of these cells gave rise to a single colony (CFU-F) (Fig. 2C).

We next examined the multi-differentiation potential of phenotypically defined MSCs. Under adipogenic and osteogenic induction conditions, single colony-derived MSCs could differentiate into adipocytes and osteoblasts as determined by Oil Red O and Alizarin Red S staining, respectively (Fig. 2B). Additionally, adipogenic (PPARγ and LPL) and osteogenic (ALP, Runx2 and OCN) marker expression was demonstrated by western blotting (Fig. 2B). These results were consistent with mesenchymal stem cell properties reported in other tissues [29]. We therefore isolated a putative population of stem cells with the methods employed in the present study, and demonstrated that the stem cells represented a putative MSC population with clonogenic renewal and multipotent differentiation capacities.

### 2. Accumulation of MSCs at the peri-implant tissue

MSCs injected into the rat tail vein were traced by the observation of samples harvested from all rats at various time points. In the present study, the injected MSCs were obtained from GFP animals [30] Thus, the transplanted MSCs could be distinguished from recipient cells. The injected MSCs were observed to accumulate at peri-implant mucosa, while no cells accumulated at gingival mucosa around natural teeth 3 days after MSC injection. Previous studies showed that MSCs specifically accumulated in injured sites with inflammation [29]. Furthermore, it was reported that soft tissue around a titanium dental implant exhibited chronic inflammation [31]. It is well known in rodents that a majority of injected MSCs were trapped in the lungs [32] and very few re-circulate. In particular, cultured MSCs attach easily to any tissue including blood vessels and lungs. Indeed, because our data and those of many previous studies show local effects of MSCs via systemic injection, this method is considered to be effective [12], [33], [34]. In this study, our data suggested that peri-implant soft-tissue inflammation strongly induced the accumulation of MSCs at an early stage.

Moreover, the location of accumulated MSCs was limited to around the apical portion of the PIE, close to connective tissue, in the early stages of wound healing. The localized MSCs were also observed in the connective tissue after tooth extraction (Fig. 3A). Because connective tissue and alveolar bone have more blood vessels than epithelial tissue [35], the inflammatory cells and the transplanted MSCs may easily accumulate in these regions.

Additionally, we determined how long MSCs remained at the inflammation site. The results showed that the isolated MSCs remained for 1 week in the tooth extraction model or 2 weeks in the implantation model. However, MSCs were reported to be present at such sites in the host mouse body for 3 days [36]. This discrepancy may be due to differences in the MSC isolation methods: Wang et al. counted the number of cells in blood, while we analyzed frozen tissue sections directly [36]. Cells accumulating at the injured site may continue to function over a period of time. Figure 3 shows that the transplanted MSCs around the implant remained at the local site twice as long as those around the tooth extraction socket. As shown in our previous study [15], because the recovery of soft tissue around the implant takes much longer than around the tooth extraction socket, the MSCs were maintained for longer in the implantation group compared with the extraction group (Fig. 3B). Therefore, the disappearance of inflammation may release the accumulated MSCs from the titanium surface or the tooth extraction site.

### 3. Distribution of laminin-332 in the peri-implant oral mucosa

Laminin-332, which mediates the adhesion of basal cells via integrin α6β4, is expressed at the interface between junctional epithelium and natural tooth [22], [37] and is predicted to be critical for the attachment of the gingival epithelial cells to substrates [38]. In our previous study, laminin-332 was implicated in the adhesion of the PIE to the dental implant [22]. Therefore, we observed the distribution of laminin-332 during PIE formation around the implant to eliminate the influence of transplanted MSCs on OE.

As previously reported, laminin-332-positive staining was apparent as a band along the implant-PIE interface in most areas except the upper portion [18]. However, the injection of MSCs extended the positive band into this upper portion. Systemic MSC application induced laminin-332 expression by the epithelial cells on the dental implant (Fig. 5). MSCs promote tissue regeneration, as indicated in Figure 4, not only by multi differentiation and proliferation, but also by cytokine expression (IGF-1, FGF, PDGF, etc.) to activate cells involved in wound healing [39]. The effect of MSCs on the epithelium after implantation or extraction was long lasting, as shown in Figure 2, although there were a small number of exotic MSCs around the wounded site in the early stages. In contrast, our previous report showed that the expression of laminin-332 on the titanium implant surface was accelerated by the specific growth factor, IGF-1, which promoted PIE formation and improved epithelial sealing around the dental implant [25]. Therefore, we suggest that the MSCs were induced to stimulate tissue regeneration by growth factors in the MSC-injected group at 4 weeks (Fig. 5).

### 4. Relationship between MSCs and OECs in co-culture conditions

Although it has been shown that MSCs clearly promoted epithelium wound healing and epithelial attachment to the implant surface (Figs. 4 and 5), the mechanism by which the transplanted MSCs activated the epithelial cells is not clear. In Figure 6, the changes in OEC adhesion and proliferation when co-cultured directly or indirectly with MSCs were shown to have a strong relationship with MSCs. As a result, in assays to evaluate the strength of OEC adherence, the adhesion of OECs was accelerated under the indirect co-culture condition (Fig. 6A). Similarly, proliferation of OECs was markedly increased only by indirect co-culture of MSCs and OECs using trans-wells (Fig. 6B). These results appeared to be inconsistent with *in vivo* data showing that injected MSCs accumulated around the implant and promoted epithelial cell attachment to the implant surface. Considering their indirect culture in trans-wells only, MSCs promoted the adhesion and proliferation of OECs on the titanium surface.

## Conclusion

In this study, systemic MSC application accelerated OE healing and PIE formation after tooth extraction and implantation, respectively. Additionally, laminin-332 expression at the adhesion structures along the implant-PIE interface was improved by MSC injection. Therefore, systemically applied MSCs may significantly improve the protection of the PIE from peri-implant inflammation.

## Supporting Information

Figure S1 **Accumulation of GFP-transgenic injected MSCs after implantation.** Around the experimental implants, there were many CD-90/CD-44 or CD-90/CD-105 double-positive cells in the mucosa. The location of accumulated MSCs was limited to around the apical portion of the PIE-like epithelial structure. However, fibroblasts isolated from GFP-transgenic rat back skin, which were injected via the tail vein similar to MSCs, did not accumulate at any site. Bar = 100 µm.
(TIFF)

Figure S2 **Accumulation of GFP-transgenic injected MSCs at various organs.** The location of accumulated MSCs, CD-90/GFP double-positive cells, was limited to around the experimental implants. However, almost all injected cells were detected in lung and a few cells were in heart, liver. Bar = 20 µm.
(TIFF)
