# Supplementary figures and images for: Therapeutic Interaction of Systemically-Administered Mesenchymal Stem Cells with Peri-Implant Mucosa
Source: PLoS One. 2014 Mar 20;9(3):e90681. doi: 10.1371/journal.pone.0090681 (PMC3961234; doi:10.1371/journal.pone.0090681)

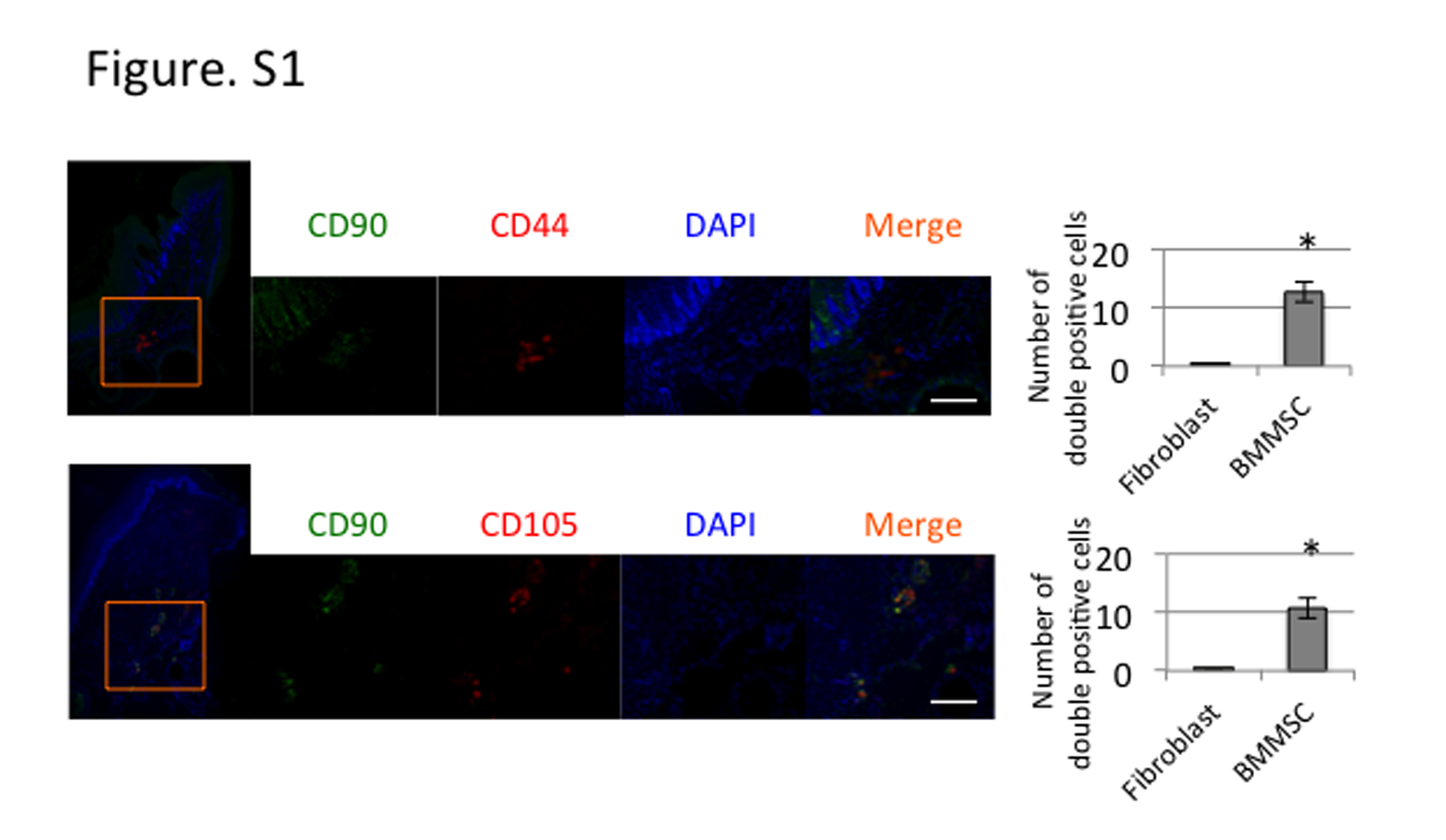

Supplement: Figure S1 — Accumulation of GFP-transgenic injected MSCs after implantation. Around the experimental implants, there were many CD-90/CD-44 or CD-90/CD-105 double-positive cells in the mucosa. The location of accumulated MSCs was limited to around the apical portion of the PIE-like epithelial structure. However, fibroblasts isolated from GFP-transgenic rat back skin, which were injected via the tail vein similar to MSCs, did not accumulate at any site. Bar = 100 µm. (TIFF) [file pone.0090681.s001.tiff]

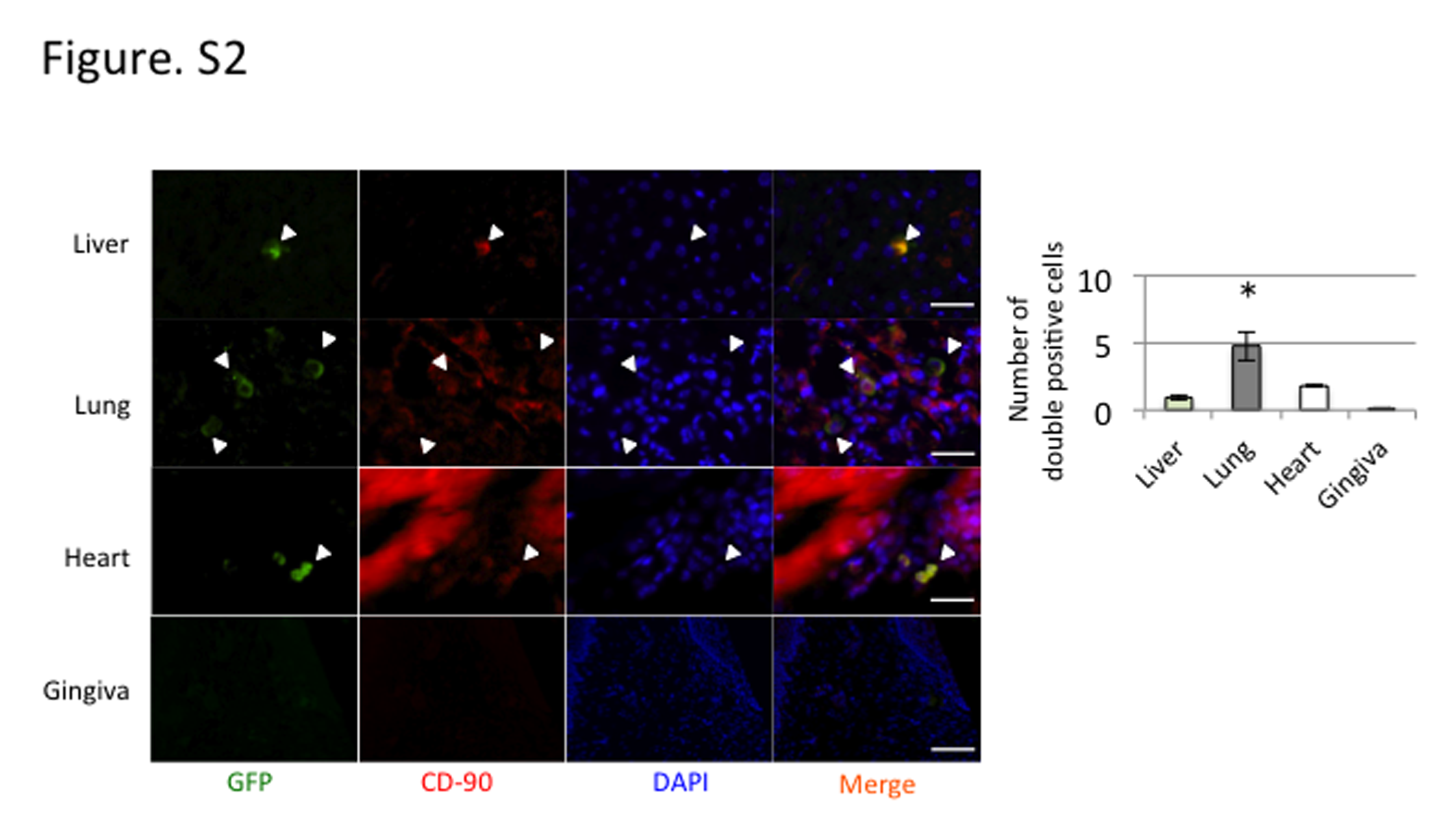

Supplement: Figure S2 — Accumulation of GFP-transgenic injected MSCs at various organs. The location of accumulated MSCs, CD-90/GFP double-positive cells, was limited to around the experimental implants. However, almost all injected cells were detected in lung and a few cells were in heart, liver. Bar = 20 µm. (TIFF) [file pone.0090681.s002.tiff]
